# HCMV Infection in a Mesenchymal Stem Cell Niche: Differential Impact on the Development of NK Cells versus ILC3

**DOI:** 10.3390/jcm9010010

**Published:** 2019-12-19

**Authors:** Ricarda Ising, Sandra Weinhold, Sabrina Bianca Bennstein, Albert Zimmermann, Özer Degistirici, Gesine Kögler, Roland Meisel, Hartmut Hengel, Jörg Timm, Markus Uhrberg

**Affiliations:** 1 Institute for Transplantation Diagnostics and Cell Therapeutics, Heinrich Heine University Düsseldorf, Medical Faculty, 40225 Düsseldorf, Germany; 2 Institute of Virology, Heinrich Heine University, University Hospital of Düsseldorf, 40225 Düsseldorf, GermanyJoerg.Timm@med.uni-duesseldorf.de (J.T.); 3 Division of Pediatric Stem Cell Therapy, Clinic for Pediatric Oncology, Hematology and Clinical Immunology, Center for Child and Adolescent Health, Heinrich Heine University Düsseldorf, Medical Faculty, 40225 Düsseldorf, Germany; 4 Institute of Virology, University Medical Center, Albert-Ludwigs-University Freiburg, 79104 Freiburg, Germany; 5 Faculty of Medicine, Albert-Ludwigs-University Freiburg, 79104 Freiburg, Germany

**Keywords:** Mesenchymal stem cells, Natural killer cell development, Human cytomegalovirus, Innate lymphoid cells, Graft-versus-host disease

## Abstract

Human cytomegalovirus (HCMV) is highly prevalent in most populations worldwide and has a major influence on shaping the human immune system. Natural killer (NK) cells are important antiviral effectors that adapt to HCMV infection by expansion of virus-specific effector/memory cells. The impact of HCMV infection on the development of NK cells and innate lymphoid cells (ILC) in general is less well understood. In this context, we have recently established a novel in vitro platform to study human NK cell development in a stem cell niche based on human bone marrow-derived mesenchymal stem cells (MSC). Here, the system was modified by infecting MSC with HCMV to study the influence of virus infection on NK/ILC development. We show that cord blood-derived hematopoietic progenitor cells are successfully differentiated into mature CD56^+^CD94^+^NKG2A^+^ NK cells on HCMV-infected MSC with significant higher anti-viral cytokine production compared to NK cells developing on non-infected MSC. Furthermore, the generation of ILC3, characterized by expression of the signature transcription factor RAR-related orphan receptor gamma (RORγt) and the production of IL-22, was strongly impaired by HCMV infection. These observations are clinically relevant, given that ILC3 are associated with protection from graft-versus-host disease (GvHD) following stem cell transplantation and HCMV reactivation in turn is associated with increased incidence of GvHD.

## 1. Introduction

Mesenchymal stem cells (MSCs) are self-renewing and multipotent mesenchymal stromal cells able to differentiate into various mesodermal cell types, such as osteoblasts, chondrocytes, and adipocytes [1]. Due to their unique features, in particular their immunoregulatory functions, MSCs are routinely used as cellular therapeutics in the clinic [2]. After birth, MSCs mainly reside within the bone marrow (BM), but are also found at extramedullary sites such as cord blood (CB), fat, and other tissues. Besides their important immunoregulatory and stem cell functions, MSCs play a critical role as component of the hematopoietic stem cells niche in the bone marrow [1]. Within the stem cell-supporting microenvironment of the BM niche, MSCs promote differentiation of early hematopoietic stem and progenitor cells (HSPC) towards myeloid and lymphoid lineages, making MSCs a valuable tool to study immune cell development in vitro [3].

One group of immune cells assumed to develop within the BM are natural killer (NK) cells [4], whereby NK cell development is also taking place at extramedullary sites [5]. NK cells are of great importance for the innate immune response as they are able to quickly and efficiently eliminate cells that have down-regulated human leukocyte antigen (HLA)-class I molecules on the cell surface, such as in damaged, malignant, and virus infected cells [6]. The recognition of these missing-self structures occurs via the cluster of differentiation (CD) 94/NKG2A heterodimer or alternatively products of the polymorphic KIR gene family [7]. NK cells can also sense inducible stress ligands on target cells via NKG2D, a signal that is able to overwrite inhibitory signals received in parallel [8]. Furthermore, target cells that are coated with IgG antibodies are recognized by NK cells via the Fc receptor CD16 that is strongly expressed on CD56^dim^ effector NK cells inducing antibody-dependent cell-mediated cytotoxicity (ADCC) [9]. Lastly, NK cell can induce apoptosis by engagement of death receptors, in particular via the Fas/Fas-Ligand system and the release of Trail [10]. NK cells are part of the Innate Lymphoid Cell (ILC) family, which is divided into three groups according to their transcription factor (TF) and cytokine profile [11]: Group 1 ILCs comprise NK cells and ILC1 expressing T-bet and IFNγ [12], ILC2 are assigned to Group 2 and characterized by expression of GATA-3 and secretion of interleukin (IL)-13 and IL-5 [13], while lymphoid tissue inducer cells and ILC3 are both members of Group 3 that express RORγt and secrete IL-17a and/or IL-22 [14,15]. A common ILC precursor (CILCP) giving rise to NK cells as well as all three ILC groups has been recently identified within human and murine BM [16,17,18]. 

In recent years it became clear that the NK cell compartment and possibly also other ILC subsets are majorly shaped by infection with human cytomegalovirus (HCMV) [19]. NK cells differentiate in response to HCMV into so-called adaptive NK cells, characterized by their high sensitivity to CD16-mediated ADCC and the upregulation of the stimulatory receptor NKG2C that recognizes HCMV-infected cells on the basis of their increased expression of HLA-E [20]. Adaptive NK cells are expanded to very high frequencies in some latently infected individuals [21]. Recent evidence suggests that these expansions are dependent on the co-expression of cognate killer cell immunoglobulin-like receptors (KIR) possibly by providing survival signals upon engagement of cognate HLA class I ligands [22]. 

HCMV is one of the most complex herpes viruses (human herpes virus 5) and has dedicated large parts of its genome to the diversion of the host immune system [23]. HCMV replicates comparatively slowly and is able to persist within the host in a latent state throughout life [24]. HCMV infection has a high prevalence world-wide, ranging from 40%–100% antibody seropositivity depending on the country of origin [25]. Whereas the symptoms during acute HCMV infection are usually mild and subclinical in the immune-competent host, it constitutes a serious threat for patients with suppressed immune system, in particular in organ and clinical stem cell transplantation (HSCT), leading to increased mortality [25]. On the other hand, HCMV reactivation was reported to decrease the risk of relapse in leukemic patients, suggesting improved tumor control due to HCMV-mediated activation of the immune system [26,27,28,29]. Hence, it appears to be of great interest to better understand how HCMV interacts and manipulates the host immune system in acute and latent infection. 

The effects of HCMV infection on human NK/ILC cell development are currently not well understood, partly because established in vitro assays for studying NK cell differentiation are based on co-culture systems utilizing murine stroma cells [30]. Since HCMV, as many other herpes viruses, is strictly host specific, murine stromal cells cannot serve as model for an HCMV-infected stem cell niche. In this context, we have recently established BM-derived MSCs as a novel human stem cell niche that efficiently and robustly support NK cell development from cord blood (CB)-derived HSPC [31]. It should be stressed that MSCs play a dual role in this context, since on the one hand they are well described to suppress NK and T cell effector functions [32] and are clinically employed as immunoregulatory cellular therapeutics [33], but on the other hand are supporting hematopoietic development in the bone marrow stem cell niche [3]. 

MSCs were previously shown to be permissive for HCMV infection [34,35]. In the present study we utilized the MSC/HSPC co-culture system as an in vitro model to analyze NK/ILC development during acute HMCV infection. To this end, we compared NK cell development from CB-derived CD34^+^ HPSCs on HCMV-infected (+HCMV) and HCMV-uninfected (−HCMV) MSCs. While NK cells were generated at comparable frequencies on -HCMV MSCs compared to +HCMV MSCs, the latter showed significantly enhanced effector functions in terms of cytotoxicity and production of the anti-viral cytokines IFN and TNF. Unexpectedly, whereas NK cells and ILC3 cells are generated at roughly similar frequencies on -HCMV MSC, ILC3 development was strongly impaired by HCMV-infected MSC. The present findings have potentially important clinical implications since ILC3 cells are playing key roles in maintaining the integrity of the gut epithelia as well as the microbiotic environment [36] and HCMV-mediated ILC3 depletion could thus contribute to increased gut pathology in graft-versus-host disease following stem cell transplantation.

## 2. Experimental Section

### 2.1. Human Samples, Cell lines and Cell Culture

CB samples were obtained from healthy volunteer donors after informed consent. This study was approved by the ethics committee of the Medical Faculty of Heinrich-Heine-University. CD34^+^ HSPCs were isolated with magnetic beads according to the manufacturer´s instructions with a two-step protocol using the Lineage Cell Depletion Kit and CD34 MicroBead Kit (Miltenyi Biotec, Bergisch Gladbach, Germany). MSCs were isolated and cultivated as previously described in detail [31]. MRC-5 fibroblasts were cultivated in the same medium as MSC. The B-lymphoblastoid HLA-E-transfected 721.221 cell line was generously provided by Prof. Khakoo, University of Southampton, UK and cultivated in RPMI (Gibco by Thermo Fischer Scientific, Waltham, MA, USA) with 10% fetal calf serum (Biochrom AG, Berlin, Germany) and 1% Penicillin/ Streptavidin (Gibco by Thermo Fischer Scientific, Waltham, MA, USA) [21]. 

### 2.2. Infection of MSC with HCMV

In all infection experiments, the HCMV strain AD169 was used that was previously shown to productively infect MSC [34,35]. The laboratory strain AD169 has a deletion of 15 kb in the UL region (reading frame 133–151), an internal repeat long (IRL) between the UL and US region (duplication within the reading frame 1–14), and a point mutation within gene 131 (ATCC; BVL, 2007). Prior to infection, MSCs were seeded for at least 24 h within 24-well plates until reaching a density of 80%–100%. The infection was performed at a multiplicity of infection (MOI) of 0.1–3 by directly adding the virus into the wells. After titration all MSC infections were done at a MOI of 0.5. To increase efficiency of viral infection, plates were centrifugated twice for 15 m at 870× *g*. Between the first and second centrifugation step, the plate was rotated 180° to ensure a homogenous infection. The plates were kept at 5% CO_2_ and 37 °C. 

### 2.3. Co-culturing MSCs/ 721.211 and HSCP 

MSCs were seeded at least 48 h prior to co-cultivation and subsequently infected with HCMV 24 h before adding HSCP (2000/well). At that time point, fresh NK1 medium was added. After 7 days, 80% of NK1 medium was replaced with NK2 medium. Depending on the proliferative rate, 50% fresh medium was added every 3–4 days and co-cultures were maintained for 28–35 days at 37 °C and 5% CO_2_. NK1 medium contained 2/3 DMEM 4.5 g/mL glucose (Gibco by Thermo Fischer Scientific, Waltham, MA, USA), 1/3 Ham’s F12 (Biochrom AG, Berlin, Germany), 20% heat inactivated human AB serum (Biochrom AG), 20 mg/L ascorbic acid (Sigma-Aldrich, St. Louis, MO, USA), 50 µmol/L ethanolamine (Sigma-Aldrich, St. Louis, MO, USA), 50 µmol/L sodium-selenit (Sigma-Aldrich), 24 µmol/L 2-mercapthoethanol (Gibco by Thermo Fischer Scientific, Waltham, MA, USA), 1% Penicillin/ Streptavidin (Gibco by Thermo Fischer Scientific, Waltham, MA, USA), 1% L-Glutamin (Gibco by Thermo Fischer Scientific, Waltham, MA, USA), 5 ng/mL IL-3 (Miltenyi Biotec, Bergisch Gladbach, Germany), 20 ng/mL IL-7 (Miltenyi Biotec, Bergisch Gladbach, Germany), 20 ng/mL SCF (Miltenyi Biotec, Bergisch Gladbach, Germany), 1000 U/mL IL-2 (Novartis), and 10 ng/mL Flt-3l (Miltenyi Biotec, Bergisch Gladbach, Germany). NK2 medium contained 2/3 DMEM 4.5 g/mL glucose, 1/3 Ham’s F12, 10% heat inactivated human AB serum, 20 mg/L ascorbic acid, 50 µmol/L ethanolamine, 50 µmol/L sodium-selenit, 24 µmol/L 2-mercapthoethanol, 1% Penicillin/ Streptavidin, 1% L-Glutamin, 10 ng/mL IL-15 (Miltenyi Biotec, Bergisch Gladbach, Germany), 20 ng/ml IL-7, 20 ng/mL SCF, 1000 U/mL IL-2, and 10 ng/mL Flt-3l. NK3 medium contained the same ingredients like NK2 except Flt3L, SCF, and IL-7. Additionally, 10 ng/mL IL-12 was added (Miltenyi Biotec, Bergisch Gladbach, Germany). 

### 2.4. Flow Cytometric Analyses 

For extracellular staining, the cells were harvested, blocked with staining buffer (1 × PBS w/o containing 2 mM EDTA and 0.5% BSA, both ROTH, Karlsruhe, Germany) and stained for 20 min with fluorescence-conjugated antibodies (see Table S1). For intracellular staining the cells were fixed and permeabilized with Fixation solution (Biolegend, San Diego, CA, USA) and stained intracellularly. For intranuclear staining, cells were fixed and permeabilized with the Foxp3 intranuclear staining kit (eBioscience, San Diego, CA, USA) and stained for the transcription factor RORC (see Table S2). 

### 2.5. Analysis of NK Cells and ILC3 Function

Cultured cells were harvested and mixed with K562 target cells at an effector/target ratio of 1:1. The CD107a assay and the staining of intracellular cytokines were performed as previously described [31,37]. For analysis of IL-22 production, cultured cells were harvested and re-stimulated with 10 ng/well of IL-1β and IL-23 for 17 h. After 1 h, Brefeldin A (2 µL of 1:10 dilution of BD GolgiPlug Protein Transport Inhibitor) and Monensin (2 µL of 2 mM, Biolegend, San Diego, CA, USA) were added. Intracellular staining was done with anti- IL22 PE/Cy7 (clone: 2G12A41, Biolegend, San Diego, CA, USA). 

### 2.6. Statistical Analyses

Statistical analyses were done with Graph Pad Prism 5 (GraphPad Software, San Diego, CA, USA. The height of the bars represents the mean ± standard error of the mean (SEM). Levels of significance were calculated using a student’s *t* test, non-parametric *t* test, and a non-parametric One-Way ANOVA, * *p*-value < 0.05, ** *p*-value < 0.01, *** *p*-value < 0.001.

## 3. Results

### 3.1. HCMV Infection and Virus Replication in Bone Marrow-Derived Human MSC 

**Figure 1:**
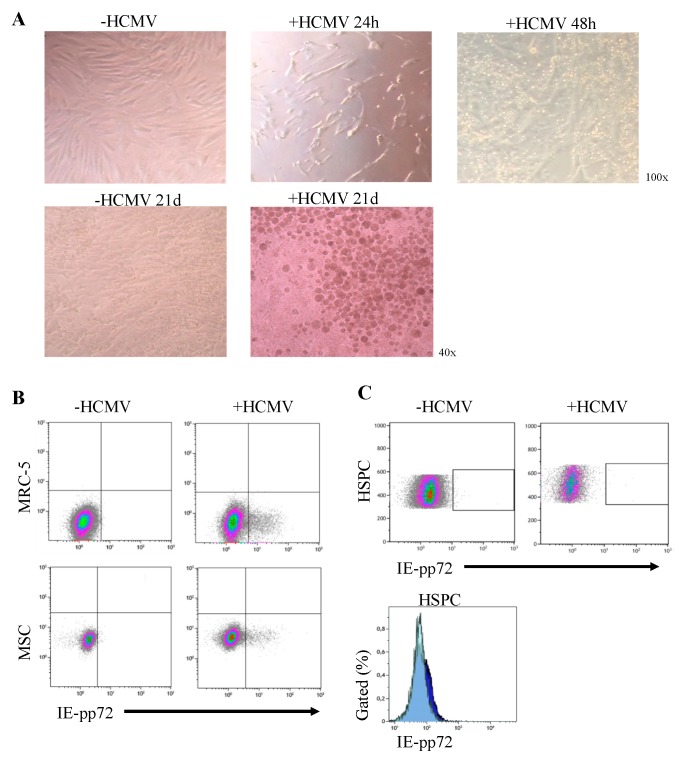
MSCs but not HSPCs are productively infected with HCMV. (**A**) Representative micrographs (upper panel: original magnification ×100, lower panel: original magnification ×40) depicting the morphology of MSCs during NK cell differentiation from day 0 to day 21 with (+HCMV) and without (-HCMV) infection. (**B**) Representative dot plots showing the expression of the HCMV-encoded protein IE-pp72 on the HCMV-infected (right) and uninfected (left) fibroblast cell line MRC-5 (upper panel), MSC (lower panel), and (**C**) HSPC (upper panel), as well as a histogram overlay of IE-pp72 expression of HSPC cultured on +HCMV (light blue) and –HCMV MSC (dark blue) after the first replication cycle (72 h). Abbreviation: MSC (mesenchymal stem cells), HCMV (human cytomegalovirus), HSPC (hematopoietic stem and progenitor cells), h (hour), d (day) IE (immediately early).

In order to establish an in vitro model to study NK/ILC development during an acute HCMV infection, conditions had to be worked out to ensure efficient infection of MSC without co-infecting HSPC while still enabling NK cell differentiation. All MSC lines utilized in this study were established from BM of children (age 1.5–8.5 years) under GMP conditions as previously described [31]. Upon infection with the HCMV strain AD169, changes in the morphology of +HCMV MSCs were already visible 24 h post-infection. Whereas -HCMV MSCs typically formed a dense cell layer with spindle shape and individual cells being clearly visible, +HCMV MSCs start to enlarge showing first signs of disintegration of the MSC layer after 48 h. Nonetheless, +HCMV MSCs were still viable even at 21 d of culture with a distinct spherical and enlarged phenotype. Control -HCMV MSCs remained in a dense cell layer with typical spindle morphology (Figure 1A).

We next wanted to determine viral gene expression within +HCMV MSCs by analyzing expression of the immediately early (IE)-pp72 protein, which is essential for the efficient induction of a productive infection cycle [38]. As positive control we used human MRC-5 fibroblasts which represent an optimal target cell for efficient HCMV replication [39]. As expected, no IE-pp72 protein was detectable within -HCMV MSCs and MRC-5, while IE-pp72 was detected within +HCMV MSCs and MRC-5 (3.86% vs. 8.02%). We aimed at a low initial infection rate to mimic the natural course of HCMV infection and spread in vivo. By minimizing infection-mediated cytotoxicity and enabling a continuous and gradual infection process of the MSC layer, niche-mediated support is provided throughout the complete NK cell differentiation process (Figure 1B). Importantly, no signs of HCMV gene expression were found in HSPC, neither by microscopic evaluation of cell shape nor by analysis of IE-pp72 protein, which was not detectable within HSPC (Figure 1C). The above data demonstrate that MSCs can be successfully infected with HCMV and that viable +HCMV MSCs can be propagated in vitro for several weeks.

### 3.2. NK Cell Differentiation in the Presence of HCMV-Infected MSC 

**Figure 2:**
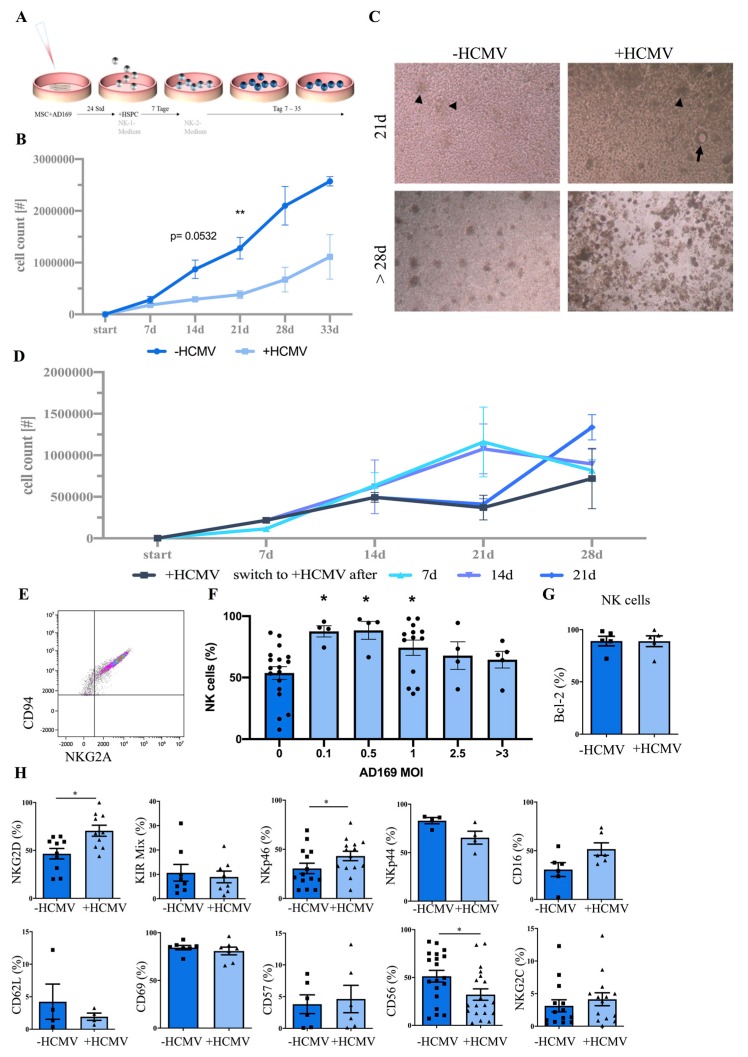
NK cell differentiation from CD34^+^ HSPC in a HCMV-infected MSC niche. (**A**) Overview of the experimental set-up: CD34^+^ HSPCs were isolated from CB and seeded onto the MSC stromal monolayers infected with HCMV (strain AD169) on 24-well plates. NK cell differentiation was induced with NK1 and NK2 media at the indicated timepoints. The cultures were analyzed for up to 35 days depending on the experiment. (**B**) Total cell numbers (per well in a 24-well plate) on -HCMV MSC (dark blue line) and +HCMV MSC (light blue line) at the indicated time points (*n* = 2–13, depending on the time point). (**C**) Representative micrographs of NK cells cultured on +/-HCMV MSCs for 21 days (upper panel) and 28 or more days (lower panel). (**D**) Plate switch experiments in which HSPC cultured on +HCMV MSCs were removed from the original +HCMV MSCs and seeded onto freshly infected +HCMV MSCs at day 7, 14, or 21. The black line represents the control condition where HSPCs were not switched to freshly infected MSCs. The dark blue line represents a switch at day 7, the purple line at day 14, and the medium blue line at day 21. (**E**) Representative dot plot of flow cytometric NKG2A and CD94 analysis enabling identification of NK cells in the cultures. (**F**) NK cell frequencies (gated on NKG2A^+^ cells) on -HCMV and +HCMV MSC in relation to different viral concentrations (MOI: multiplicity of infection; representing the ratio between virus particles and target cells) at day 21 (*n* = 4). (**G**) Quantification of BCL-2 expression in NK cells in infected (+HCMV, MOI 0.5, AD169) and uninfected (-HCMV) cultures (*n* = 4). (**H**) Flow cytometric quantification of typical NK cell surface receptors on NK cells (gated on NKG2A^+^CD56^+^) developed on +/-HCMV MSC (MOI 0.5, AD169) after 21 days of culture: NKG2D (*n* = 9), KIR-mix (comprising KIR mAbs for 2DL1, 2DL2, 2DL3, and 3DL1) (*n* = 8), NKp46 (*n* = 14), NKp44 (*n* = 4), CD16 (*n* = 5), CD62L (*n* = 6), CD69 (*n* = 3), CD57 (*n* = 6), CD56 (*n* = 19), and NKG2C (*n* = 14). The heights of the bars represent the mean ± standard error of the mean (SEM). Levels of significance were calculated with a mixed-effects analyses with a post-test comparing conditions (B/D), a non-parametric One-Way ANOVA (Kruskal–Wallis) with a post-test comparing NK cells generated on -HCMV MSC with NK cell frequencies with different AD169 MOIs (**F**), and with a student’s *t* test (G/H), * *p*-value < 0.05.

Next, given that MSC are permissive to HCMV infection as well as virus replication, we explored the possibility to utilize HCMV-infected MSC as stroma cell support to enable NK cell differentiation studies in an in vitro model of acute infection. To this end, HSPCs were isolated from CB and seeded onto the respective stromal monolayers. In the +HCMV condition, MSCs were infected with HCMV 24 h prior to co-cultivation as outlined in the experimental scheme (Figure 2A). Culture conditions including dosing and timing of cytokines IL-3, IL-7, IL-15, SCF, and IL-2 were adapted from previous studies using murine feeder cells [31,40]. Whereas in the first week of culture, no difference in cell proliferation was detected between +HCMV and -HCMV MSCs, after two weeks cell numbers were already lower on +HCMV MSCs (∅ 2.9 × 10^5^ cells/ per well) compared to uninfected MSC (∅ 8.7 × 10^5^ cells/ well) with a significant difference seen at day 21 (+HCMV MSC ∅ 3.8 × 10^5^ cells/ per well, -HCMV MSC ∅ 1.2 × 10^6^ cells/ well, *p*-value: 0.0075). Nevertheless, in both conditions a continuous increase in total cell numbers was observed translating into a 500-fold expansion on +HCMV MSC versus 2500-fold on -HCMV MSC (Figure 2B). 

We next wanted to find out if the decreased proliferation rate on +HCMV MSCs was a direct effect of virus infection on the developing NK cells or rather due to compromised MSC-mediated support. Regarding the latter, a dense -HCMV MSC layer with formation of developing NK cell clusters was observed after three weeks, whereas +HCMV MSC layers were disrupted with surviving MSC exhibiting a typical spherical and enlarged cell shape (Figure 2C), similar to the experiments without HSPC co-culture shown above (Figure 1A). To analyze this more thoroughly, plate switch experiments were performed, where developing cells were transferred onto layers of freshly infected +HMCV MSCs at different time points. Indeed, increased proliferation rates were observed when replacing infected MSC after 2 or 3 weeks (Figure 2D) suggesting that the infected MSCs were gradually losing supportive capacity over time leading to the observed decrease in cell proliferation. Of note, after four weeks, independent of the condition, the MSC layers were typically gone, which was probably due to the activity of cytotoxic effector NK cells that are present at large numbers at this late stage of culture, recognizing allogeneic MSCs as targets.

Next, in order to sustain the supportive capacity of MSC in the co-culture system as long as possible, we aimed to minimize the cytopathic effects of HCMV infection on MSC by determining the optimal virus concentration. We observed the highest frequencies of NK cells, determined as cells expressing the bona fide NK cell receptor CD94/NKG2A (Figure 2E), at MOIs from 0.1 to 1.0, while higher MOIs led to decreased NK cell frequencies (Figure 2F). Consequently, a MOI of 0.5 was selected for further experiments, representing the optimal compromise between low cytopathic effect and high infection rate of MSC. Finally, to evaluate if NK cells generated on +HCMV MSC exhibited decreased cell survival, developing NK cells were stained for expression of b-cell lymphoma 2 (BCL-2), representing an anti-apoptotic factor that is well established to play a key role for NK cells survival [41]. However, no difference in BCL-2 expression was observed between NK cells generated on +HCMV or -HCMV MSCs (Figure 2G). Altogether, the above data suggest that the MSC/HSPC co-culture system represents a robust in vitro platform to study the influence of acute HCMV infection on NK cell differentiation.

### 3.3. HCMV Infection Leads to Enhancement of NK Cell Effector Functions 

**Figure 3:**
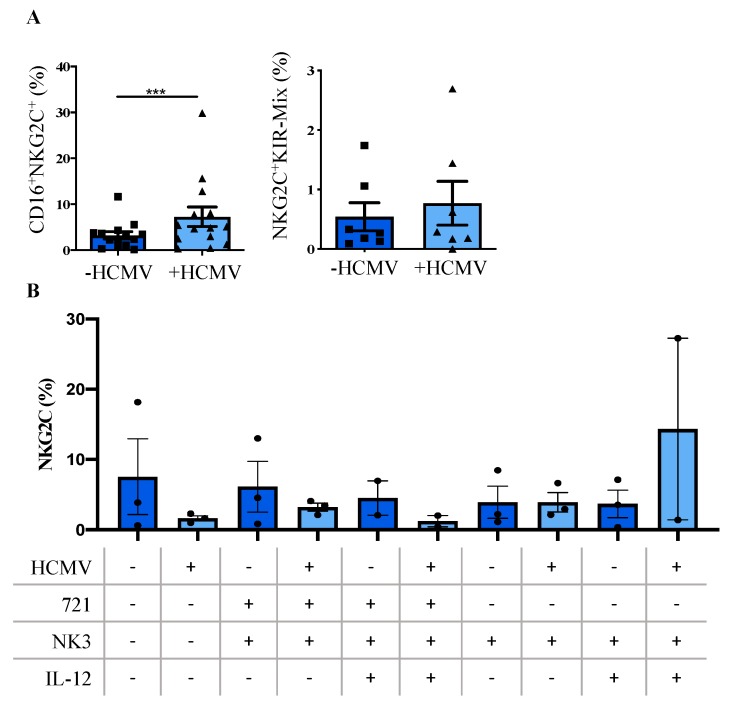
HCMV+ MSC encourage a significant up-regulation within CD16^+^NKG2C^+^ NK cells, but without significant NKG2C^+^ NK expansion. (**A**) Representative dot plots for CD16^+^NKG2C^+^ NK cells and NKG2C^+^KIR-Mix^+^ of +HCMV MSC (light blue, boxes) and -HCMV MSC (dark blue, triangles) cultures gated on CD34^-^CD3^-^ cells (*n* = 7–13). (**B**) NKG2C frequencies of NK cell at day 25 following plate switch experiments starting on +HCMV MSC (light blue) and -HCMV MSC (dark blue) cultures at day 18 and subsequent plate switch to irradiated HLA-E transfected 721.221 cells, either with or without IL-12, NK3 medium alone or IL12 alone (*n* = 2–3). The height of the bars represents the mean ± SEM. Levels of significance were calculated using a student’s *t* test (a) and a One-Way ANOVA (b). *** *p*-value < 0.001. Abbreviations: CD (Cluster of differentiation), HCMV (human cytomegalovirus), IL (interleukin), within (B) + (added), - (not added).

We next compared the phenotypic and functional properties of NK cells generated on +HCMV and -HCMV MSC. As outlined above, most NK cells generated in the MSC/HSPC co-culture system expressed the CD94/NKG2A heterodimer, which is the predominant inhibitory receptor for recognition of HLA class I on CD56^bright^ NK cells but is also expressed, albeit at lower frequency, on the more mature CD56^dim^ NK cells. Whereas CD56^dim^ NK cells constitute the main cytotoxic effector subset, CD56^bright^ NK cells predominantly produce cytokines but are mostly non-cytotoxic [42]. Unfortunately, it is not possible to distinguish between the two major NK cell subsets on the basis of CD56 expression in vitro, since CD56 is quickly upregulated in culture thereby blurring the distinction between the two subsets. Thus, to evaluate the frequency of mature effector NK cells we analyzed the expression of selected KIR genes (mixture of KIR2DL1, KIR2DL3/2DL3, and KIR3DL1-specific mAbs), of CD16, and of CD57, all representing established hallmarks of mature and terminally differentiated CD56^dim^ effector NK cells. None of these maturation markers exhibited significant changes although CD16 and CD57 showed a tendency to be increased in the course of infection. As outlined in Figure 2H, no significant differences were also detected for the activation-sensitive molecules CD62L, CD69, and NKp44. In contrast, significant expression differences were found for the key stimulatory NK cell receptors NKG2D and NKp46, as well as for the adhesion molecule and NK cell marker CD56. Of note, there was also no expansion detectable for NKG2C^+^ NK cells within +HCMV MSC cultures (Figure 2H). However, we observed a significant up-regulation of NKG2C^+^ NK cells in the CD16^+^ subset within HCMV-infected cultures but without concomitant up-regulation in KIR expression (Figure 3A). We next wanted to find out if this population constituted a putative direct precursor of KIR^+^ adaptive NK cells capable to expand as observed in vivo following acute HCMV infection [21,22]. To this end, we performed switch-plate experiments, in which emerging NK cells were transferred at day 18 from conventional MSC cultures onto 721.221 cells transfected with HLA-E, constituting one of the strongest stimuli for expansion of adaptive NK cells [21]. However, again no NKG2C expansion was observed under these conditions (Figure 3). 

**Figure 4:**
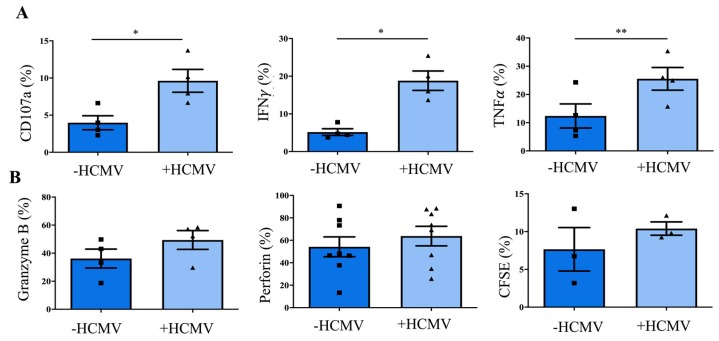
Cytotoxic granule mobilisation and cytokine secretion is enhanced in NK cells generated on HCMV-infected MSCs. Cells were gated on NKG2A^+^CD56^+^ NK cells generated on -HCMV or +HCMV MSC and the indicated molecules analysed by flow cytometry after day 28–33. MSCs were infected with AD169 at an MOI of 0.5. (**A**) Quantification of CD107a, IFNγ, and TNFα after co-culture with K562 for 6h at an effector/ target ratio of 10:1 (*n* = 4). (**B**) Quantification of steady-state expression of Granzyme B, Perforin, and killing ability measured by a CFSE assay after co-culture with K562 for 6h at an effector/ target ratio of 10:1 (*n* = 3–8). The heights of the bars represent the mean ± SEM. Levels of significance were calculated by a student’s t test. * *p*-value < 0.05, ** *p*-value < 0.01.

We next performed an evaluation of HCMV-mediated changes in NK cell effector functions. Firstly, NK cells generated on +HCMV MSC exhibited significantly stronger surface expression of CD107 in response to K562 target cells (Figure 4; *p*-value: 0.0473). Notably, differences in CD107 mobilization, representing an established read-out for target cell-induced granule mobilization to the cell surface [43] were not associated with differences in intracellular granzymes and perforin expression, representing the main effector components of cytotoxic granules [44]. In line with this no difference in killing capacity was observed. Secondly, the frequency of NK cells producing key NK cell effector cytokines, in particular IFNγ, and to a lesser extend also TNFα was also significantly increased in NK cells from +HCMV MSC cultures (*p*-values: IFNγ: 0.0041; TNFα: 0.0175). Together, the present data suggest enhancement of NK cell effector functions towards anti-viral immunity in an HCMV-infected hematopoietic stem cell niche. 

### 3.4. Differential Impact of HCMV Infection on the Development of NK Cells versus ILC3

**Figure 5:**
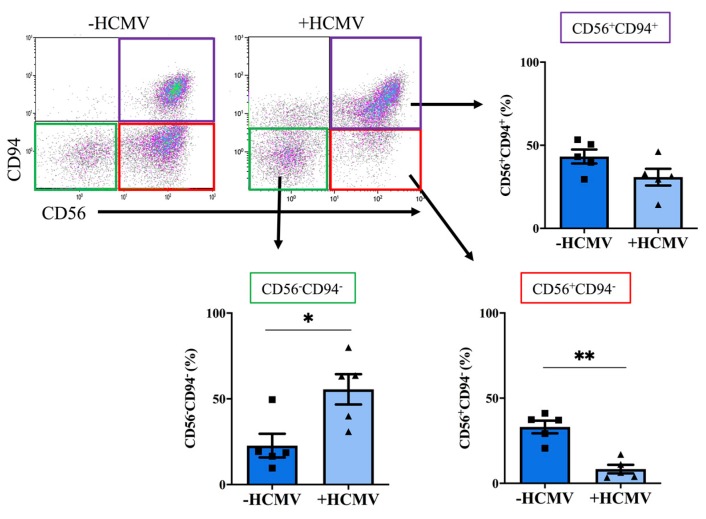
HCMV-infection of MSC suppresses development of CD56^+^CD94^-^ cells. Representative dot plots for CD56 and CD94 expression (gated on CD34^-^CD117^-^) from cultures generated on -HCMV MSCs (left hand side) and +HCMV MSCs (right hand side; MOI 0.5, AD169) with corresponding gating of representative populations: CD94^-^CD56^-^ (green box), CD94^+^CD56^+^ (representing NK cells, purple box), and CD94^-^CD56^+^ (red box) after 28–33 days of co-culture (*n* = 5). Frequency changes within the individual populations generated on +HCMV MSC (light blue bar) and –HCMV MSC (dark blue bar) are shown. The heights of the bars represent the mean ± SEM. Levels of significance were calculated by a non-parametric unpaired t test (Mann–Whitney U). * *p*-value < 0.05, ** *p*-value < 0.01.

It was previously reported that differentiation of HSPC in NK cell-promoting conditions, besides NK cells, also leads to the development of ILC3, an innate lymphoid cell type characterized by expression of the transcription factor RORγt and the production of IL-22 [45]. In this regard, in the present experiments, we regularly noted the generation of a subset of CD56^+^ cells that did not express CD94/NKG2A, which is at first glance compatible with an ILC3 phenotype (Figure 4). Unexpectedly, we observed a significant decline of this CD56^+^CD94^-^ subset when developed on +HCMV MSC compared to -HCMV MSC (∅ 8.4% vs. ∅ 33.1%; *p*-value: 0.008). Moreover, concomitant with the decline of CD56^+^CD94^-^ cells, significantly higher frequencies of CD56^-^CD94^-^ cells were observed on +HCMV MSC compared to -HCMV MSC cultures (∅ 55.5% vs. ∅ 22.7%; *p*-value: 0.032). In contrast, no significant changes were observed for NK cells in both conditions (+HCMV MSC: ∅ 43.2% vs. +HCMV MSC: ∅ 30.8%) (Figure 5).

**Figure 6:**
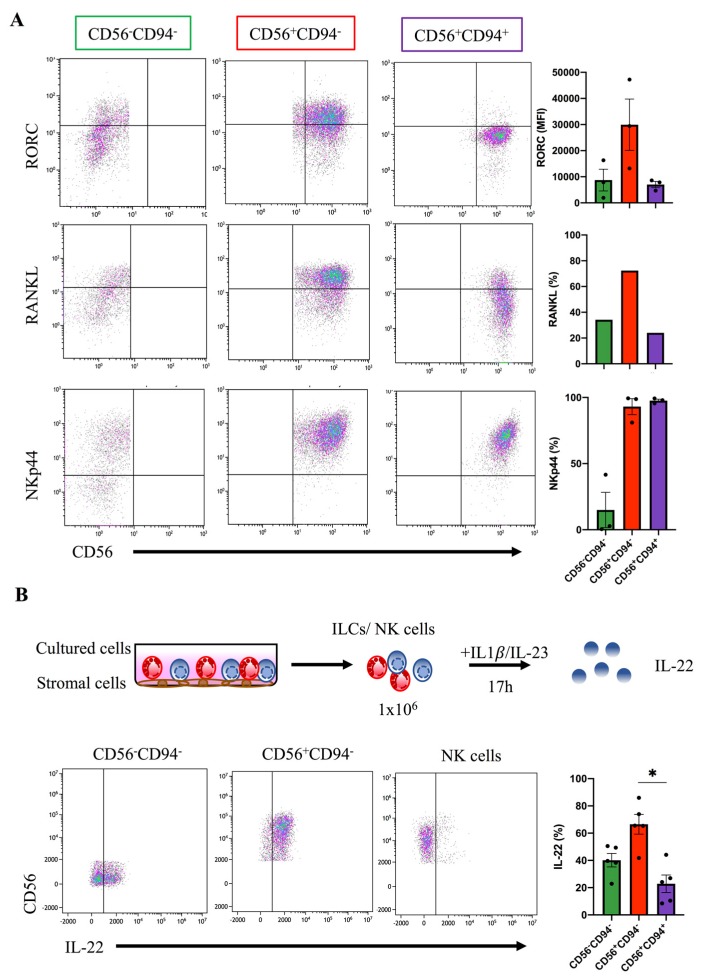
The HCMV-sensitive CD56^+^CD94^-^ subset phenotypically and functionally resembles ILC3. MSC/HSPC co-cultures were analysed after day 28–33. (**A**) The three major cell subsets generated within the +/-HCMV cultures were gated as shown in Figure 5 and analysed for expression of the indicated molecules. Representative dot plots are shown for RORγt (upper panel), RANKL (middle panel), and NKp44 (lower panel) against CD56 for NK cells generated on -HCMV MSC. Bars on the right-hand side indicate the MFI (RORγt) and frequencies (RANKL and NKp44) for all three subsets (*n* =1–3). (**B**) Cultures generated on -HCMV MSCs were restimulated with IL1β and IL-23 (10 ng per well each) for 17 h and analysed for IL-22 expression. Representative dot plots (CD56 versus IL-22) and quantification of IL-22 expression for CD56^-^CD94^-^ (green bars), CD56^+^CD94^-^ (red bars), and NK cells (CD56^+^CD94^+^, blue bars) are shown. The heights of the bars represent the mean ± SEM. Levels of significance were calculated by a non-parametric One-way ANOVA, * *p*-value < 0.05.

Next, the CD56^+^CD94^-^ subset was characterized more closely to determine if it phenotypically and functionally corresponds to ILC3 cells. Indeed, intranuclear staining for RORγt expression, representing a signature transcription factor of ILC3, revealed strong expression in CD56^+^CD94^-^ but not NK cells (CD56^+^CD94^+^) and moderate expression in the CD56^-^CD94^-^ subset. Furthermore, high expression was also found for receptor activator of NF-κB ligand (RANKL) as previously described for ILC3 [11], representing a factor involved in survival, proliferation, and differentiation [46]. Moreover, NKp44 expression was high in CD56^+^CD94^-^ and CD56^+^CD94^+^, consistent with previous observations that NKp44 is expressed on a subset of ILC3 mainly observed within tissues [15] but also on in vitro stimulated NK cells [47] (Figure 6A). Finally, we analyzed the production of IL-22, representing a key effector function of NKp44^+^ILC3 [14,15,45]. When differentiation cultures were restimulated with IL-1β and IL-23, the majority of CD56^+^CD94^-^ exhibited IL-22 production whereas only few NK cells were stimulated by this treatment. The CD56^-^CD94^-^ subset again showed an intermediate response (IL-22 secretion: CD56^+^CD94^-^: 66.5%; CD56^-^CD94^-^: 40.2%; NK cells: 22.8%, *p*-value: 0.011) (Figure 6B). Altogether, the present data strongly suggest that the CD56^+^CD94^-^ subset indeed represents ILC3 and that their development is specifically inhibited in the HCMV-infected MSC niche. HCMV infection would thus lead to a bias in differentiation towards preferential development of NK cells. 

## 4. Discussion

CMV infection profoundly influences various components of the immune system and is one of the major extrinsic factors of immune variation [19]. In the case of NK cells, acute CMV infection leads to the expansion of terminally differentiated, so-called adaptive NK cells able to specifically eliminate infected cells reminiscent of the expansion of virus-specific memory T cells. Whereas the CMV-induced modulation of NK cell effector responses is well described in humans and mice [22,48,49], its impact on NK cell development is less well understood. In this study we were able to successfully generate NK cells from CD34^+^ HSPC on HCMV-infected MSC. We could demonstrate that HMCV infection does not impair NK cell development, but instead significantly enhances anti-viral effector functions of the newly generated NK cells. Of note, HCMV infection did not induce substantial expansion of NKG2C^+^ NK cells suggesting that the HCMV-infected mesenchymal stem cell niche was not providing the proper signals to induce the development of adaptive NK cells. Although it is currently not known at which developmental stage and at which site they are initially generated, it is common believe that NKG2C^+^ adaptive NK cells are terminally differentiated cells originating from already mature CD56^dim^ NK cells, which are mostly located in the circulation. Thus, the MSC-based niche would preferentially support development of NK cells up to the stage of mature NK cells but would not support or might even suppress the generation of terminally differentiated NKG2C^+^ NK cells given the powerful immunoregulatory properties of MSC towards effector T and NK cells [33].

The present data reveal that the chosen conditions not only support development of NK cells but also the generation of ILC3. The latter are distinguished from NK cells by the lack of NK cell receptors such as CD94/NKG2A and KIR, the expression of the transcription factor RORγt, and the production of IL-22 [11,14,45]. We show that the CD56^+^CD94^-^ subset, which is abundantly generated in the HSPC/MSC co-cultures, are ILC3 based on intranuclear staining for RORγt, the production of IL-22 upon stimulation with IL-1β and IL-23, and the expression of NKp44 and RANKL. This is reminiscent of previous observations employing murine stroma cells as stem cell niche, which similarly supported the generation of NK cells and ILC3 from CB-derived HSPC [45,50]. However, the present protocol to our knowledge constitutes the first in vitro platform to generate mature, IL-22-producing ILC3 in fully human culture conditions. Notably, we demonstrate that the large majority of CD56^+^CD94^-^ cells generated on MSCs were indeed mature RORγt ^+^, IL-22-producing ILC3, whereas this was only the case for a minor subset when generated on murine stroma cells [45]. Of note, co-culturing of MSCs with ex vivo isolated human tonsillar ILC3s was shown to enhance ILC3 proliferation and IL-22 production [51]. 

The robust generation of large amounts of mature ILC3 in the HSPC/MSC co-culture system constitutes a versatile feature for future cellular therapies employing ILC3. Of note, in contrast to NK cells that can be readily isolated from peripheral blood, ILC3 are primarily present in secondary lymphoid and intestinal tissues [14,15,52,53], making their isolation for cellular therapies logistically challenging or even impossible. Moreover, it was recently shown that ILC3-like cells in the periphery are mostly ILC progenitors lacking the capacity to rapidly produce IL-22 [54]. Thus, the present system, employing MSC established in good manufacturing standard (GMP) conditions, provides a versatile option to generate ILC3 as potential cellular therapeutics in clinical settings such as inflammatory diseases of the gut. An important role for ILC3 in maintaining gut integrity and barrier function is also supported by mouse studies showing inhibition of bacterial translocation through intestinal epithelia and their involvement in the clearance of infection with pathological bacteria strains such as Escherichia coli and Salmonella typhimurium as well as with helminths (reviewed in [55]). Importantly, whereas NKp44^+^ ILC3, as generated in the present in vitro system, play important roles in maintaining gut integrity by producing IL-22, a pathological role is suggested for NKp44^-^ ILC3s that produce IL-17 and are found to be enriched in Crohn’s Disease [56,57]. Of note, in an experimental murine GvHD model, IL-22 secreting ILC3 were identified to protect intestinal stem cells [58]. Moreover, when further analyzing the reconstitution capacity of human ILC after allogenic HSCT in leukemic adults, an increase of donor NKp44^+^ILC3 was observed, which was associated with reduced susceptibility to GvHD [59]. 

Our study demonstrates that ILC3 development is profoundly dysregulated in a HCMV-infected hematopoietic stem cell niche. Furthermore, HCMV reactivation is associated with an increased risk for development of GvHD [60] following HSCT. It is thus tempting to speculate that a block in ILC3 development occurring in acute HCMV infection would aggravate the risk of GvHD by selectively depleting IL-22-producing ILC3. However, there are no clinical data available so far comparing reactivation of HCMV following HSCT with the frequency and protective function of NKp44^+^ ILC3. The underlying mechanisms leading to the HCMV-mediated inhibition of ILC3 development remain to be elucidated. One possible mechanism would be that HCMV-encoded factors modulate expression of surface ligands or soluble molecules on infected MSC leading to down-regulation of transcription factors such as RORγt that are essential for ILC3 but not NK cell development. On the other hand, ILC3 development in the MSC niche might not be directly impaired, but expansion of mature ILC3 might be regulated by NK cells, which were shown here to be potent producers of antiviral cytokines when generated on +HCMV MSC and thus might also exert cytotoxic functions. Finally, direct killing of ILC3 by HCMV appears to be unlikely as mature lymphocytes are generally not infected by HCMV. Of note, the depletion of ILCs, especially of ILC3, in the course of virus infection has already been described for human immunodeficiency virus (HIV) and simian immunodeficiency virus (SIV) infection [61,62,63]. However, so far studies only reported the loss of ILC3 during an infection but not the impairment of ILC3 development. 

In summary, the present MSC/HSPC co-culture system provides a unique in vitro platform to study the generation of ILCs including NK cells and ILC3 in the setting of acute HCMV infection. We report profound virus-induced changes in the composition of the ILC subsets, supporting NK cell development and increasing their functionality while strongly decreasing the generation of ILC3. The question in how far reactivation of HCMV is associated with a decline of ILC3 frequency in the setting of clinical stem cell transplantation is currently open and merits further investigation. In general, the present platform enables the generation of large numbers of ROR t ^+^, IL-22-producing ILC3 in a GMP-compatible process, which could be exploited for future cellular therapies of clinical conditions characterized by the loss of gut barrier integrity including acute bacterial infections, GvHD, or inflammatory bowel disease.

## Supplementary Materials

The following are available online at https://www.mdpi.com/2077-0383/9/1/10/s1, Table S1: Antibodies used for extracellular staining, Table S2: Antibodies for intracellular/ nuclear staining.
