# Supplementary material for: HCMV Infection in a Mesenchymal Stem Cell Niche: Differential Impact on the Development of NK Cells versus ILC3
Source: J Clin Med. 2019 Dec 19;9(1):10. doi: 10.3390/jcm9010010 (PMC7027004; doi:10.3390/jcm9010010)
Supplement: Supplementary file 1 [file jcm-09-00010-s001.pdf]

Table S1: Antibodies used for extracellular staining

**Specificity**	**Fluorochrome**	**Clone**	**Manufacturer**
CD3	APC	UCHT1	Biolegend
CD3	APC/Cy7	UCHT1	Biolegend
CD3	BV510™	UCHT1	Biolegend
CD3	FITC	UCHT1	Biolegend
CD3	Pacific blue	UCHT1	Biolegend
CD3	PE/Cy5	UCHT1	Biolegend
CD3	PE/Cy7	UCHT1	Biolegend
CD14	FITC	HCD14	Biolegend
CD14	PE	HCD14	Biolegend
CD16	APC/Cy7	3G8	Biolegend
CD16	FITC	3G8	Biolegend
CD16	PE/Cy7	3G8	Biolegend
CD16	PE	3G8	Beckman Coulter
CD19	FITC	HIB19	Biolegend
CD19	PE	HIB19	Biolegend
CD34	BV510™	581	Biolegend
CD34	PE	581	Biolegend
CD38	PE/Cy5	HIT2	Biolegend
CD56	APC	HCD56	Biolegend
CD56	APC/Cy7	HCD56	Biolegend
CD56	BV421™	HCD56	Biolegend
CD56	PE/Cy5	HCD56	Biolegend
CD56	PE/Dazzle™ 594	HCD56	Biolegend
CD56	PE	N901	Beckman Coulter
CD69	PE/Pazzle™5 94	FN50	Biolegend
CD94	APC	DX22	Biolegend
CD94	FITC	DX22	Biolegend
CD94	PE	DX22	Biolegend
CD117	BV421™	104D2	Biolegend
CD158a,h	FITC	HP-MA4	Biolegend
CD158b1,b2,j	FITC	DX27	Biolegend
CD158c1	PE	DX9	Biolegend
NKG2A	APC	Z199	Beckman Coulter
NKG2A	PE/Cy7	Z199	Beckman Coulter
NKG2A	PE	REA110	Miltenyi Biotech
NKG2D	PE	1D11	Biolegend
NKp46	BV510™	9E2	Biolegend
NKp44	PE/Cy5	Z231	Beckman Coulter
RANKL	PE	MIH24	Biolegend
CD107a	APC/Cy7	H4A3	Biolegend

Table S2: Antibodies for intracellular/ nuclear staining

Specificity	Fluorochrome	Clone	Manufacturer
Bcl2	AF700™	100	Biolegend
Granzyme B	Pacific Blue	GB11	Biolegend
Granzyme B	FITC	GB11	BD Pharmingen
IEpp72	AF488™	8B1.2	Millipore
IFNγ	FITC	B27	Biolegend

IFNγ	PE/Cy7	B27	Biolegend
Perforin	PE	dG9	Biolegend
TNFα	APC/Cy7	Mab11	Biolegend
IL-22	PE/Cy7	2G12A41	Biolegend
RORC (intranuclear)	PE	AFKJS-9	eBioscience
